# Neurodevelopmental disorder caused by an inherited novel *KMT5B* variant: case report

**DOI:** 10.3325/cmj.2023.64.334

**Published:** 2023-10

**Authors:** Ljubica Odak, Katarina Vulin, Ana-Maria Meašić, Lara Šamadan, Ana Tripalo Batoš

**Affiliations:** 1 Department of Medical and Laboratory Genetics, Endocrinology and Diabetology with daily care unit, Children’s Hospital Zagreb, Zagreb, Croatia; 2 European Reference Network – Intellectual disability, Tele Health, Autism and Congenital Anomalies; 2 Department of Pediatric Radiology, Children’s Hospital Zagreb, Zagreb, Croatia

## Abstract

Neurodevelopmental disorders are a large group of disorders that affect ~ 3% of children and represent a serious health problem worldwide. Their etiology is multifactorial and includes genetic, epigenetic, and environmental causes. Mounting evidence shows the importance of genetic causes, especially genes involved in the central nervous system development. As recently discovered, the *KMT5B* gene is related to abnormal activities of the enzymes that regulate histone activity and gene expression during brain development. Pathogenic *KMT5B* gene variants lead to autosomal dominant, intellectual developmental disorder 51 (OMIM # 617788). Also, reports on patients with additional features suggest that the *KMT5B* gene alterations lead to multisystem involvement. Here, we report on a male patient with a severe neurodevelopmental disorder caused by a novel *KMT5B* gene variant inherited from his mother. The patient had severe intellectual disability, absent speech, marked autistic behavior, attention deficit hyperactivity disorder, and different clinical features, including thoracic scoliosis, dysmorphic facial features, and tall stature. In contrast, his mother, with the same *KMT5B* variant, had mild intellectual disability and some autistic traits (stereotype hand movement). We elucidated pathogenetic mechanisms that could influence phenotype characteristics. Our findings emphasize the importance of a comprehensive clinical and molecular approach to these patients in order to provide optimal health care.

Neurodevelopmental disorders (NDs) represent a large group of disorders characterized by intellectual impairment, and language, social, behavioral, and motor deficits. Mounting evidence shows the importance of genetic causes, especially genes involved in synaptic organization, and plasticity and brain development (1,2). In recent years, special attention has been dedicated to investigating genes involved in histone modification that could alter the expression of genes involved in normal brain development. Pathogenic *KMT5B* gene variants are a rare cause of intellectual disability, and there is a lack of literature data about patients with these variants. Here, we report on a novel *KMT5B* gene variant in a patient with a severe neurodevelopmental disorder.

## CASE REPORT

We present a case of a 16-year-old male patient with a severe neurodevelopmental disorder and additional features. His mother had a mild intellectual disability and some autistic traits (stereotype hand movement), while his father had schizophrenia. There were no consanguinity or NDs in other family members.

The patient was born at term (birth weight 3500 g, birth length 52 cm, Apgar score 10/10), without perinatal complications. His somatic growth was normal, but he had hypotonia and delayed motor, speech, and language development. He started walking at the age of 18 months and he never developed speech. He underwent an early intervention program, and speech, occupational, and physical therapy.

Clinical evaluation at the age of two did not reveal visceral anomalies. Routine biochemical, hematological, and metabolic tests were unremarkable. He did not have visual impairment, while auditory testing showed signs of central hearing impairment.

**Figure 1:**
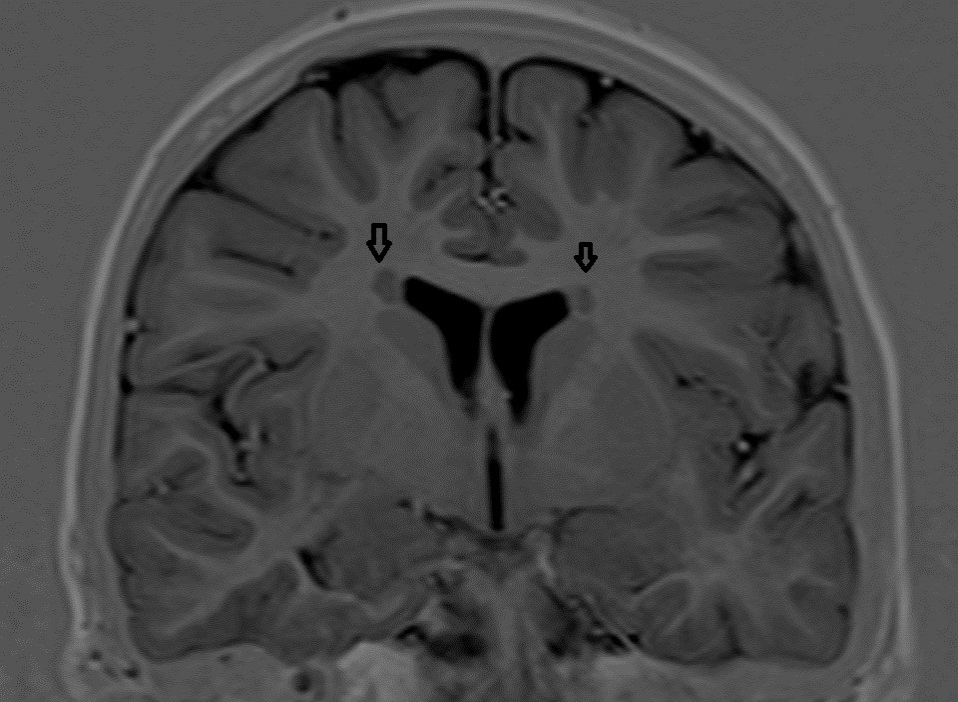
Brain magnetic resonance imaging in a patient with a novel *KMT5B* variant (coronal section): periventricular nodular heterotopia marked with arrows.

At the age of three, the developmental assessment confirmed a global developmental delay. At the age of 10, he developed grand mal seizures that required antiepileptic drug therapy with carbamazepine. Brain magnetic resonance imaging showed bilateral periventricular nodular heterotopia adjacent to the nucleus caudatus and an asymmetry of the nucleus caudatus itself (Figure 1). In adolescence, he developed severe thoracic scoliosis requiring surgical treatment.

**Figure 2:**
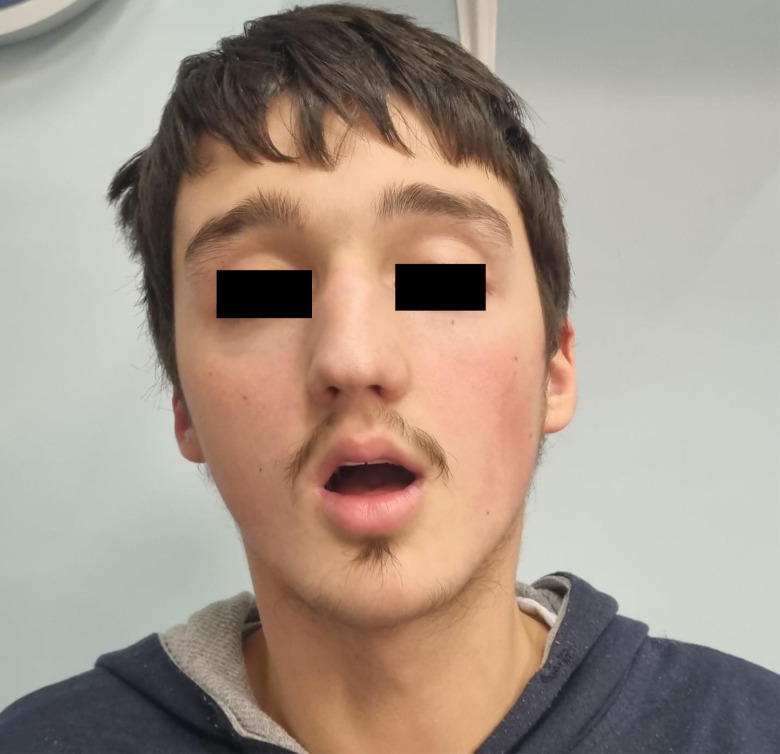
Dysmorphic features in a patient with a novel *KMT5B* variant: long face, thick eyebrows, upslanted palpebral fissures, wide and elongated nasal bridge, narrow mouth.

A detailed evaluation at the age of 16 showed severe intellectual disability, tall stature, thoracic scoliosis, and dysmorphic features: a long face, thick eyebrows, upslanted palpebral fissures, a wide and elongated nasal bridge, and a narrow mouth (Figure 2). He had never developed sphincter control and speech. His autistic behavior manifested as a lack of social skills, poor emotional control, and attention deficit hyperactivity disorder.

**Figure 3:**
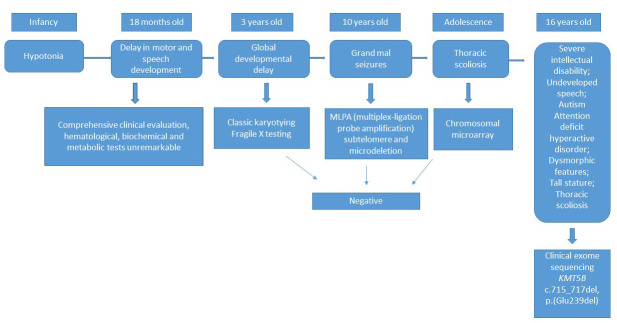
The timeline of major clinical and diagnostic medical history information. MLPA - multiplex-ligation probe amplification.

**Figure 4:**
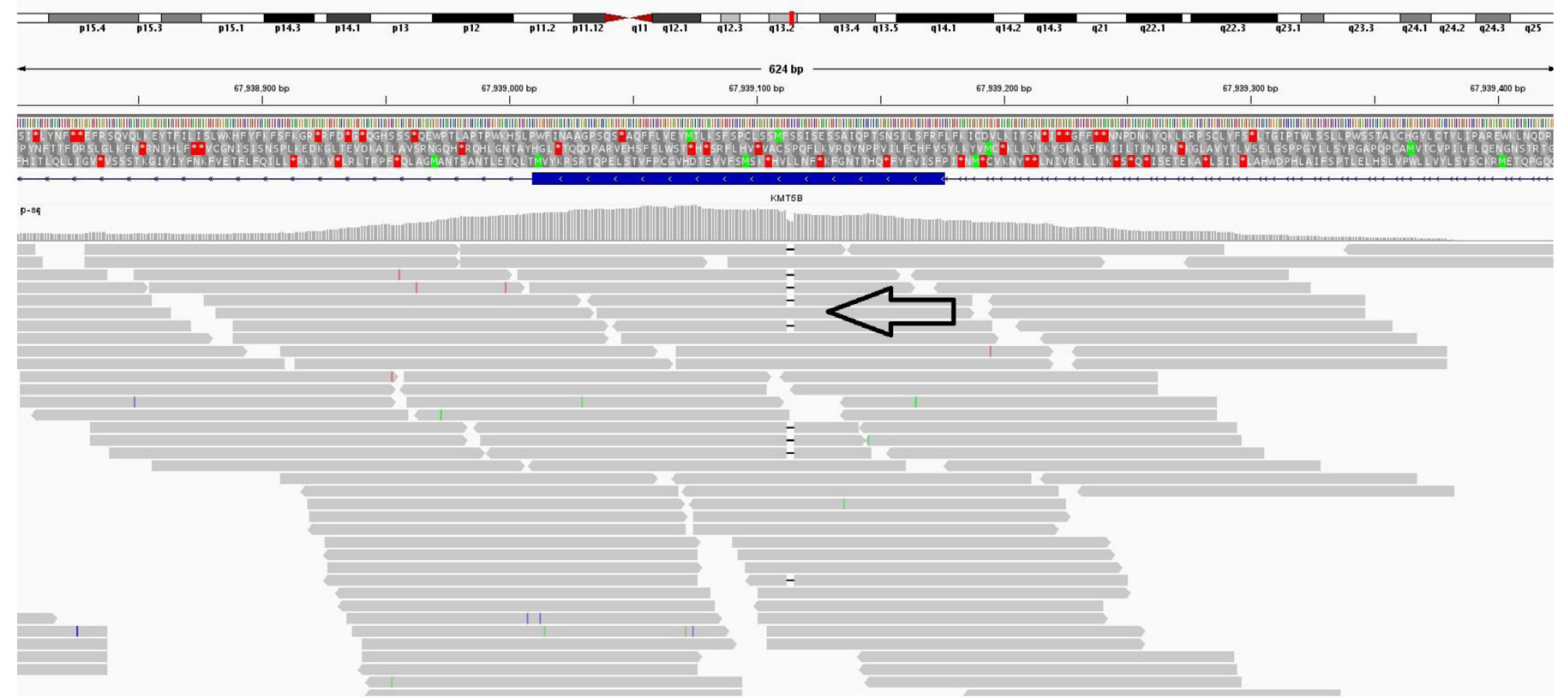
A heterozygous in-frame deletion was detected in exon 7 of the *KMT5B* gene at the position NM_017635.4 c.715_717del p.(Glu239del).

The clinical genetic workup included classical and molecular (chromosomal microarray analysis) karyotyping, multiplex-ligation probe amplification (MLPA) subtelomere and microdeletion analysis, and fragile X testing (Figure 3). All tests were negative. He also underwent clinical exome sequencing (CES) with the TruSight One panel (Illumina, San Diego, CA, USA). The analysis was performed with the Human Phenotype Ontology codes related to phenotype features. The data were interpreted according to the following databases: Human Genome Mutation Database, USC Genome Browser, Decipher, PubMed, ClinVar, Varsome, Franklin, Online Mendelian Inheritance in Man (OMIM), Leiden Open Variation Database, and the GenHGMDome Aggregation Database (gnomAD) (access date: June 16, 2023). Clinical exome sequencing revealed an in-frame deletion of three nucleotides (CTC) at c.715_717 in the *KMT5B* gene (reference sequence NM_017635.4), which resulted in a deletion of glutamic acid (Glu) in the codon p.239 (Figure 4). The same variant was present in his mother.

## DISCUSSION

The complex genetic architecture of NDs comprises hundreds of genes involved in the pathogenesis of autism and intellectual disability (3). The *KMT5B* gene (OMIM *61088) encodes the SUV420H2 protein, lysine methyltransferase, which affects the site K20 on histone protein H4. Growing evidence suggests the importance of H4K20 methylation in brain development, prenatal neurogenesis, and neuronal migration (4). Recent studies have shown strong expression of this methyltransferase in the developing nervous system and a role in the pathogenesis of NDs. Pathogenic variants in *KMT5B* lead to autosomal dominant intellectual developmental disorder 51 (OMIM # 617788), a new entity described a few years ago (5). The largest study focusing on this entity included 43 patients presenting with global developmental delay, epilepsy, intellectual disability, macrocephaly, autism spectrum disorder, and congenital anomalies (6). Most of these variants are *de novo* variants (90%), missense, nonsense, frame-shift, splice-site, and rarely partial gene deletions or single amino-acid deletions. Our patient had a previously unreported in-frame deletion variant at the position c.715_717, which led to glutamic acid deletion. According to the American College of Medical Genetics and Genomics classification, it is a likely pathogenic variant (PM1 – moderate, PM4 – moderate, PM2 – supporting, PP5 – supporting). Furthermore, protein coding length changes as a result of an in-frame deletion in the *KMT5B* gene, and this variant is not located in a repeat region. So, according to UniProt, protein KMT5B_HUMAN in domain 'SET' has 18 missense/in-frame variants (8 pathogenic, 10 uncertain, and no benign variants), which qualifies it as a moderately pathogenic variant. In addition, the variant was not found in gnomAD genomes or exomes. Our patient had common KMT5B-related features: intellectual disability, epilepsy, autism, and dysmorphic features (Figure 2). However, congenital heart disease and macrocephaly, observed in 30- 60% of the patients, were absent (6). Our patient had hypotonia in infancy, motor delay, and poor skeletal muscle mass, all of which were absent in his mother. Animal models found a possible role of the *KMT5B* gene in muscle development and maturation (7). A mouse model with an altered *KMT5B* gene showed reduced muscle mass and body weight, especially in males (7). Another animal model found different neurobehavioral phenotypes in male and female mice (8). Interestingly, our patient’s mother had mild intellectual disability and autistic features, without dysmorphic features, and she is almost independent in her daily activities and self-care. There is a need for further studies that could elucidate the observed phenotype and sex differences.

Human and mouse *KMT5B* gene expression models showed altered TGF beta signaling in mutant variants and abnormalities in extracellular matrix metabolism, which could be related to neocortex development (6). Our patient had periventricular nodular heterotopia not previously described in KMT5B patients, a possible new feature of KMT5B-related disorder (Figure 1). However, as the patient’s father had schizophrenia, we cannot exclude the effect of paternally inherited genes and pathways (9).

In conclusion, we reported on a patient with a complex neurodevelopmental phenotype and a new KMT5B mutation and elucidated pathogenetic mechanisms that could influence the phenotype characteristics. Our findings emphasize the importance of a comprehensive clinical and molecular approach to these patients in order to provide optimal health care.
